# The links between wood traits and species demography change during tree development in a lowland tropical rainforest

**DOI:** 10.1093/aobpla/plad090

**Published:** 2023-12-27

**Authors:** Andrés González-Melo, Juan Manuel Posada, Jacques Beauchêne, Romain Lehnebach, Sébastian Levionnois, Géraldine Derroire, Bruno Clair

**Affiliations:** Biology Department, Faculty of Natural Sciences, Universidad del Rosario, Avenida carrera 24 # 63C-69. Bogotá, Colombia; Biology Department, Faculty of Natural Sciences, Universidad del Rosario, Avenida carrera 24 # 63C-69. Bogotá, Colombia; CIRAD, UMR Ecologie des Forêts de Guyane (EcoFoG), AgroParisTech, CNRS, INRAE, Université des Antilles, Université de Guyane, 97337, France; CNRS, Laboratory of Botany and Modeling of Plant Architecture and Vegetation (UMR AMAP), 34398 Montpellier, France; CNRS, UMR Ecologie des Forêts de Guyane (EcoFoG), AgroParisTech, CIRAD, INRAE, Université des Antilles, Universite de Guyane, Kourou, 97310France; CIRAD, UMR Ecologie des Forêts de Guyane (EcoFoG), AgroParisTech, CNRS, INRAE, Université des Antilles, Université de Guyane, 97337, France; CNRS, UMR Ecologie des Forêts de Guyane (EcoFoG), AgroParisTech, CIRAD, INRAE, Université des Antilles, Universite de Guyane, Kourou, 97310France; Laboratoire de Mécanique de Génie Civil (LMGC), CNRS, Université de Montpellier, 34000, France

**Keywords:** Demographic rates, tree size, tropical trees, ontogeny, wood traits

## Abstract

One foundational assumption of trait-based ecology is that traits can predict species demography. However, the links between traits and demographic rates are, in general, not as strong as expected. These weak associations may be due to the use of traits that are distantly related to performance, and/or the lack of consideration of size-related variations in both traits and demographic rates. Here, we examined how wood traits were related to demographic rates in 19 tree species from a lowland forest in eastern Amazonia. We measured 11 wood traits (i.e. structural, anatomical and chemical traits) in sapling, juvenile and adult wood; and related them to growth and mortality rates (MR) at different ontogenetic stages. The links between wood traits and demographic rates changed during tree development. At the sapling stage, relative growth rates (RGR) were negatively related to wood specific gravity (WSG) and total parenchyma fractions, while MR decreased with radial parenchyma fractions, but increased with vessel lumen area (*V*_A_). Juvenile RGR were unrelated to wood traits, whereas juvenile MR were negatively related to WSG and axial parenchyma fractions. At the adult stage, RGR scaled with *V*_A_ and wood potassium concentrations. Adult MR were not predicted by any trait. Overall, the strength of the trait-demography associations decreased at later ontogenetic stages. Our results indicate that the associations between traits and demographic rates can change as trees age. Also, wood chemical or anatomical traits may be better predictors of growth and MR than WSG. Our findings are important to expand our knowledge on tree life-history variations and community dynamics in tropical forests, by broadening our understanding on the links between wood traits and demography during tree development.

## Teaser

We found that the set of wood traits associated with both growth and survival rates changed during tree development in a lowland tropical rainforest. This suggests that tree size, or age, should be considered to better understand how traits shape demography.

## Introduction

The main goal of trait-based ecology is to understand how traits are related to species demography (Shipley *et al.* 2016). This is of central importance for understanding life-history variations and mechanisms of community assembly (Poorter *et al.* 2010; Wright *et al.* 2010), as well as to improve dynamic global vegetation models (e.g. Worthy and Swenson 2019). Although significant advances have been made in the last decades in explaining the links between traits and demographic rates (e.g. Poorter *et al.* 2010; Wright *et al.* 2010; Hérault *et al.* 2011; Aubry-Kietz *et al.* 2015; Visser *et al.* 2016), the predictive power of most traits on species demography is generally low (e.g. Visser *et al.* 2016; Yang *et al.* 2018). Two main reasons may explain this pattern. First, research on this topic has focussed largely on a few relatively easy-to-measure traits, which may not fully capture some plant functions (Yang *et al.* 2018). This leads to the question of whether other traits more mechanistically linked to plant functions, despite being relatively more difficult to measure, can be better predictors of species demographic rates (Poorter *et al.* 2010; Russo *et al.* 2010; Fan *et al.* 2012). Second, most previous studies on trait-demography relationships have considered either only one stem size class or have averaged broad stem size classes (e.g. Russo *et al.* 2010; Wright *et al.* 2010), overlooking size-related variations in both traits and performance (e.g. Visser *et al.* 2016; Rungwattana and Hietz 2017). Therefore, it remains unclear to what extent the effects of traits on demography change during tree development (Iida *et al.* 2014).

**Figure 1.**
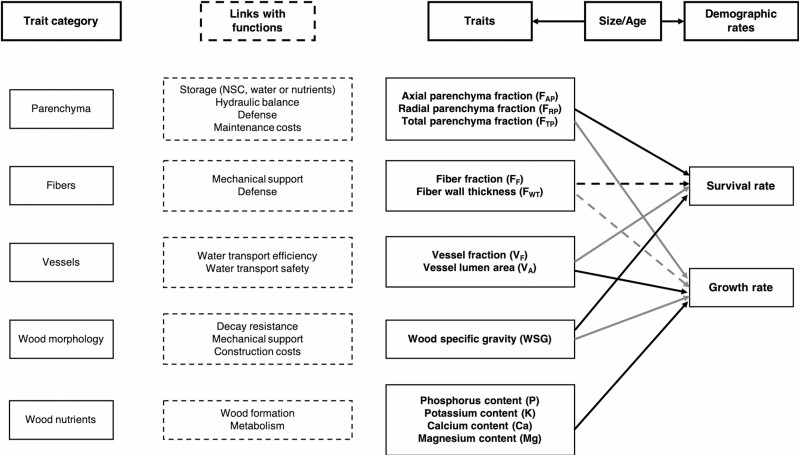
Conceptual framework showing theoretical and realized associations among wood traits and demographic rates. Black and white arrows represent positive and negative associations, respectively. Continuous arrows are theoretical associations supported by our results (see Figs 2, 3 and 4 for details), while dashed arrows are hypothesized, although nor realized, links between traits and demographics rates.

A number of studies have shown that wood-specific gravity (WSG) can predict, to some extent, species demographic rates (Chave *et al.* 2009; Poorter *et al.* 2010; Wright *et al.* 2010; Hietz *et al.* 2016; Visser *et al.* 2016). In general, WSG is negatively related to mortality and diameter growth rates (Poorter *et al.* 2008; Wright *et al.* 2010; but see Russo *et al.* 2010; see Fig. 1). Yet, specific gravity is an emergent property of wood that is affected, in angiosperms, by the fractions and morphologies of fibres, vessels and parenchyma cells (e.g. Zieminska *et al.* 2013, 2015). The main functions of these cell types are related to mechanical strength, water transport and storage, respectively (e.g. Carlquist 2001). Therefore, the relationships of WSG with growth and mortality rates (MR) may be better understood by considering wood anatomy (e.g. Poorter *et al.* 2010; Russo *et al.* 2010; see Fig. 1).

The anatomical traits underlying the relationship between WSG and diameter growth rates are relatively well studied (e.g. Russo *et al.* 2010; Fan *et al.* 2012; Hietz *et al.* 2016; Fig. 1). For example, low-WSG species grow fast in part because they generally have lower fibre fractions and/or thinner fibre walls (Zieminska *et al.* 2013), which may reduce stem construction costs (King *et al.* 2006). They also build wider vessels which favour high xylem hydraulic conductivity and support high leaf-photosynthetic carbon gain (Santiago *et al.* 2004; Poorter *et al.* 2010; Hietz *et al.* 2016). In addition, tree growth may also depend on wood nutrient concentrations (Martin *et al.* 2014; Fig. 1). For instance, physiological studies have shown that calcium (Ca), potassium (K) and magnesium (Mg) are involved in wood formation, particularly during cell expansion and differentiation (Ache *et al.* 2010; Fromm 2010). Phosphorous (P), on the other hand, plays critical role in plant metabolism as it is a structural component in ribonucleic acid (RNA), and acts as a metabolic energy unit in adenosine triphosphate (ATP, Jiang *et al.* 2019), among others. Furthermore, nutrient addition experiments in lowland tropical forests have shown that tree growth is commonly limited by soil P (Vitousek 1984; Turner *et al.* 2018) or K availability (Wright *et al.,* 2011, 2018). Thus, there are good reasons to think wood nutrient concentrations should be related to growth rates. Yet, the extent to which wood nutrients influence growth is unclear, especially in diverse tropical forests, as studies examining the links between wood nutrient concentrations and growth rates in tropical trees have been scarce and show seemingly contradictory results (Martin *et al.* 2014; Heineman *et al.* 2016).

On the other hand, it is well known that species with higher WSG tend to have lower MR (e.g. Wright *et al.* 2010; Visser *et al.* 2016). However, the anatomical mechanisms driving this relationship are still unclear (but see Poorter *et al.* 2010; Osazuwa-Peters *et al.* 2017; Fig. 1). This is due to the fact that similar values of WSG can be the product of different wood anatomies (e.g. Zieminska *et al.* 2015) and also, and more importantly because tree mortality can be simultaneously influenced by different processes (Russo *et al.* 2010; Hietz *et al.* 2016; Osazuwa-Peters *et al.* 2017). For instance, the lower MR that characterize high-WSG species may be due to high fibre fractions and thicker fibre walls that may increase structural strength (Poorter *et al.* 2010) and resistance to pathogen attacks (Alvarez-Clare and Kitajima 2007), and/or due to low xylem turgor loss points that might counteract the adverse effects of water deficits on living cells (Santiago *et al.* 2018).

In addition, there are possible links between xylem parenchyma cells and species mortality, but they remain poorly understood (Poorter *et al.* 2010; Morris *et al.* 2016*a*; Fig. 1). For instance, a higher allocation to axial (AP) and radial (RP) parenchyma cells could reduce mortality (Poorter *et al.* 2010), because these cells store non-structural carbohydrates (NSC) and nutrients (Plavcová and Jansen 2015; Kotowska *et al.* 2020), which might enable faster recovery from disturbances (Kitajima and Poorter 2007; Martin *et al.* 2014), or can provide an active response to xylem infections and mechanical damage (Carlquist 2001; Morris *et al.* 2016*b*, 2019). The effects of parenchyma cells on MR may depend, to some extent, on their stem cross-sectional fractions (Poorter *et al.* 2010; Morris *et al.* 2016*b*). While total parenchyma fractions (i.e. AP + RP) tend to be lower in high-WSG species (e.g. Fortunel *et al.* 2013; Zieminska *et al.* 2015), AP (Gonzalez-Melo *et al.*, unpubl. res.) or RP (Zheng and Martinez-Cabrera 2013) fractions can scale positively with WSG. This suggests that AP and RP cells may have different patterns of associations with WSG and MR. However, very few studies have formally examined the possible links between xylem parenchyma cells and MR across tropical tree species (but see Poorter *et al.* 2010).

As wood traits (e.g. Martin and Thomas 2013; Rungwatanna and Hietz 2017) and demographic rates (e.g. Hérault *et al.* 2011; Visser *et al.* 2016) can vary substantially with tree size, it is likely that trait-demography links may change accordingly (Fig. 1). For instance, seed size and stature are important to predict seedling performance, while WSG seems to be the best predictor of the demography of small, but not of large trees (Lida *et al.* 2014; Visser *et al.* 2016). Similarly, in a recent study conducted in a tropical semi-deciduous forest, Osazuwa-Peters *et al.* (2017) found that species demography was more strongly related to wood anatomical traits, such as cell fractions, in small than in large trees (Osazuwa-Peters *et al.* 2017). However, it is unclear if this trend holds true for other forest types. To our knowledge, no previous studies have examined the extent to which the relationships between wood anatomical traits and demographic rates change during tree development in lowland tropical humid forests.

Here, we studied how wood traits (i.e. WSG, and wood anatomical and chemical traits) are related to growth and MR in 19 tree species from a lowland forest in eastern Amazonia. In particular, we wanted to answer the following questions: (i) How are wood traits related to growth and MR? We hypothesized that diameter growth rates will increase with wood nutrient concentrations (i.e. P, K, Mg and Ca) and xylem water transport capacity (i.e. vessel lumen area), and decrease with stem construction costs (i.e. higher WSG, fibre fraction or fibre wall thickness (*F*_WT_)). We also predicted that MR will be negatively related to traits linked to stem structural strength (i.e. WSG, fibre fraction and *F*_WT_) and either to AP or RP fractions. (ii) Do the links between wood traits and demographic rates vary with tree size? We expected that the traits associated with growth and mortality would change during tree development and that the trait-demography links would be weaker at later ontogenetic stages.

## Materials and Methods

### Study site

The research was conducted in the Paracou research station, a lowland tropical rainforest located in northern French Guiana (https://paracou.cirad.fr/; 5° 18ʹN, 52° 55ʹW). At Paracou, the mean annual temperature is 28.4 °C, and annual rainfall averages *c.* 3000 mm with a marked dry season occurring between August and November, and a distinct rainy season from March to June (Wagner *et al.* 2011). Dominant families in the forests of Paracou include Fabaceae, Lecythidaceae, Sapotaceae and Chrysobalanaceae (Hérault *et al.* 2011). The landscape is characterized by moderate hills separated by narrow streams (Ferry *et al*. 2011), and soils are strongly P-limited (Grau *et al.*, 2017).

### Species and sampling

We selected 19 tree species that represent a broad gradient of variation in shade tolerance, ranging from pioneers to understory or canopy shade tolerants (Table 1). These species also spanned a wide range of WSG and wood anatomical traits (Table 2). We sampled 75 large trees (i.e. >10 cm diameter at breast height (DBH)), with two to five individuals per species (Table 1). Stem discs were collected, at breast height (*c*. 1.3 m), from previously cut-down trees. All wood samples were collected in Paracou, except for *Schefflera morototoni*, *Cecropia obtusa* and *Miconia tschudyoides*, which were collected in nearby secondary forests.

**Table 1.** Study species, family, number of trees sampled (*n*), mean diameter at breast height (DBH) of sampled individuals and ecological guilds for 19 Amazonian tree species from a lowland forest in eastern Amazonia. Ecological guilds were assigned based on Favrichon (1994), Uriarte *et al.* (2005), Scotti *et al.* (2010) and Bossu *et al.*, (2016).

Species	Family	*n*	DBH (cm)	Ecological guild	Reference
*Bagassa guianensis*	Moraceae	5	25.1	Light-demanding/Canopy	Bossu *et al.* (2016)
*Cecropia obtusa*	Urticaceae	3	16.1	Light-demanding/Understory	Favrichon (1994)
*Dicorynia guianensis*	Fabaceae	5	18.2	Semi hade-tolerant/Canopy	Favrichon (1994)
*Eperua falcata*	Fabaceae	5	19.5	Semis hade-tolerant/Canopy	Favrichon (1994)
*Eperua grandiflora*	Fabaceae	3	12.4	Semis hade-tolerant/Canopy	Favrichon (1994)
*Eschweilera coriacea*	Lecythidaceae	3	16.3	Shade-tolerant/Canopy	Favrichon (1994)
*Eschweilera sagotiana*	Lecythidaceae	2	17.8	Shade-tolerant/Canopy	Favrichon (1994)
*Hirtella glandulosa*	Chrysobalanaceae	2	15.3	Shade-tolerant/Understory	Favrichon (1994)
*Lecythis persistens*	Lecythidaceae	5	16.3	Shade-tolerant/Understory	Favrichon (1994)
*Licania alba*	Chrysobalanaceae	4	14.7	Shade-tolerant/Canopy	Favrichon (1994)
*Miconia tschudyoides*	Melastomataceae	2	13.2	Light-demanding/Understory	Favrichon (1994)
*Oxandra asbeckii*	Annonaceae	4	14.6	Shade-tolerant/Understory	Favrichon (1994)
*Parkia nitida*	Fabaceae	4	18.5	Light-demanding/Canopy	Favrichon (1994)
*Parkia velutina*	Fabaceae	3	12.4	Light-demanding/Canopy	Favrichon (1994)
*Recordoxylon speciosum*	Fabaceae	3	12.3	Semi-shade-tolerant/Canopy	Favrichon (1994)
*Schefflera morototoni*	Araliaceae	3	21.2	Light-demanding/Canopy	Uriarte *et al.* (2005)
*Sextonia rubra*	Lauraceae	3	22.4	Semi-shade-tolerant/Canopy	Scotti *et al.*(2010)
*Swartzia panacoco*	Fabaceae	3	12.5	Shade-tolerant/Canopy	Favrichon (1994)
*Virola michelii*	Myristicaceae	3	20.5	Light-demanding/Canopy	Favrichon (1994)

**Table 2.** Summary characteristics of wood traits measured on sapling, juvenile and adult wood for 19 tree species from a lowland tropical forest in eastern Amazonia. Mean, standard deviation (SD) and range of variation are shown.

Trait	Abbrev.	Unit	Sapling wood			Juvenile wood			Adult wood		
			Mean	SD	Range	Mean	SD	Range	Mean	SD	Range
Wood specific gravity	WSG	unitless	0.60	0.23	0.21–0.97	0.63	0.22	0.23–0.92	0.67	0.15	0.36–0.92
Fibre wall thickness	*F*_WT_	μm	4.10	1.86	1.05–7.87	4.44	1.88	1.48–7.73	4.80	1.83	2.01–7.71
Fibre fraction	*F*_F_	unitless	65.1	11.2	47.4–84.3	63.9	10.9	41.8–79.5	60.7	9.04	41.2–72.8
Vessel lumen area	*V*_A_	mm^2^	0.010	0.05	0.002–0.02	0.017	0.08	0.003–0.03	0.019	0.09	0.002–0.03
Total parenchyma fraction	*F*_TP_	unitless	21.1	10.4	8.55–14.6	28.2	10.1	10.6–41.5	29.9	9.34	15.5–49.0
Axial parenchyma fraction	*F*_AP_	unitless	13.2	10.3	0.46–32.4	13.0	8.45	0.84–30.8	13.5	8.11	1.93–32.8
Radial parenchyma fraction	*F*_RP_	unitless	13.1	5.79	4.30–22.8	15.1	5.99	7.35–26.6	16.3	6.04	7.70–28.8
Wood phosphorous	*P*	μg g^−1^							65.1	31.3	20.6–153.7
Wood calcium	Ca	μg g^−1^							2152	2059	281–7239
Wood potassium	Mg	μg g^−1^							162.5	164.2	19.4–643.5
Wood magnesium	K	μg g^−1^							359.2	306.0	63.2–1269
Relative growth rate	RGR	mm·mm yr^−1^	0.83	1.04	0.01–3.71	0.98	0.59	0.39–2.57	0.26	0.17	0.07–0.70
Mortality rate	MR	% yr^−1^	3.62	6.14	0.61–23.7	2.10	2.09	0.23–7.29	0.82	1.66	0.1–7.27

As wood structure is conserved as trees age, ontogenetic shifts in wood structure can be analysed by measuring radial (i.e. from pith to bark) changes in wood traits. Consequently, in each stem disc, radial segments were cut and then divided into three radial sections: (i) 1–5 cm, (ii) 5–10 cm and (iii) >10 < 50 cm [see Supporting Information—Fig. S1]. Hereafter, we refer to these radial sections as sapling, juvenile and adult wood, respectively. It is important to note that here we used stem diameter to define these three ontogenetic stages (i.e. sapling, juvenile and adult wood). Yet, we acknowledge that this categorization of ontogenetic stages can differ from others found in the literature, and may not always reflect real ontogeny because species can attain maturity at different stem diameters (e.g. Wright *et al.* 2005).

### Trait measurements

#### Wood specific gravity.

In each sapling, juvenile and adult wood section [see Supporting Information—Fig. S1], samples of *c*. 2 × 2 × 1 cm were cut every 1 cm. In these wood samples, we measured fresh volume and dry mass. Fresh volume was measured with the water displacement method, and dry mass was obtained after drying the segments at 103 °C to a constant mass for 72 h. WSG per sample was defined as dry mass over fresh volume (Kollman and Coté 1968). Then, mean WSG values for sapling, juvenile and adult wood were calculated by averaging values from the 1-cm samples. As the deposition of wood extractives (WE) in heartwood or sapwood can increase wood dry mass and, thus, alter WSG values (e.g. Lehnbach *et al.* 2019), it is possible that the links between WSG and demographic rates may be affected by the concentration of WE. To examine the effects of WE on the WSG-demography associations, we recalculated sapling, juvenile and adult WSG (i.e. WSG_REC_) of five species (*Bocoa prouacensis*, *Virola michelii*, *Sextonia rubra*, *Bagassa guianensis* and *Dicorynia guianensis*) by removing the effect of WE concentrations on wood dry mass, and run analyses (see below) using WSG_REC_ values. We obtained WE concentrations from both published (Amusant *et al.* 2014) and unpublished studies (Rodrigues 2010).

#### Wood anatomy.

In sapling and juvenile wood, anatomical analyses were conducted on one wood sample (*c.* 2 × 2 × 1 cm); whereas in adult wood, anatomy was analysed every 1.5 cm until reaching the bark, and an averaged value was calculated [see Supporting Information—Fig. S1]. To characterize wood anatomy, cross-sectional surfaces were sanded using a polishing machine with 1200-grit diamond discs, and then samples were cut with a GLS-1 sledge microtome to get a plane surface. Photographs were taken at 5–10 × objective lenses using a reflected light (episcopic) microscope (BFMX, Olympus, Tokyo, Japan), equipped with a digital camera (Canon EOS T6i; Canon Inc., Tokyo, Japan). For each wood sample, between 10 and 20 partially focussed images were taken and were then combined using Helicon Focus (Helicon Focus Ltd., Kharkov, Ukraine). From these anatomical images, vessel lumen area (*V*_A_, mm^2^) and cross-sectional area fractions of fibres (*F*_F_), vessels (*F*_V_, lumen + wall), axial (*F*_AP_), radial (*F*_RP_) and total parenchyma cells (*F*_TP_ = *F*_AP_ + *F*_RP_), were measured.

To calculate cell fractions and *V*_A_, wood cell types were manually coloured using Photoshop (Adobe Systems Incorporated, USA), and traits were calculated automatically using the batch function in the software ImageJ (https://imagej.nih.gov/ij/). *F*_WT_ (μm) was measured by taking photographs at 50–100× objective lenses using a laser microscope (VK 8850, Keyence). To do so, each anatomical image was divided into 4 equal sections, and 8 pairs of fibres were randomly selected in each section, for a total of 32 pairs of fibres per image. To obtain *F*_WT_, double wall thickness was measured and then divided by two using ImageJ. In total, we measured eight wood anatomical traits (Table 2).

#### Wood nutrient concentrations.

We measured wood nutrient concentrations in both sapling and adult wood [see Supporting Information—Fig. S1]. Yet, as wood nutrients tend to be reabsorbed from inner to outer wood, during heartwood formation, in several of our study species (A. Gonzalez-Melo, *et al.,* unpub. res.), nutrient concentrations from sapling wood were not considered reliable for this study. Therefore, here we only considered values of wood nutrient concentrations from adult wood. Wood nutrient concentrations were estimated on the sample closest to the bark. Whenever possible, nutrient analyses were conducted on the same wood samples that were used to calculate WSG and anatomical traits. To calculate wood nutrient concentrations, wood samples were ground and dry-ashed at 550 °C for 1 h, and then the ash was dissolved in HNO_3_ (1 M). Wood base cations and *P* were calculated using inductively coupled plasma-optimal emission spectrometry (ICP-OES) on an Optima 7300 DV (Perkin-Elmer Ltd., Shelton, CT, USA), with apple leaves (NIST 1515) as reference samples.

#### Demographic rates.

To calculate adult (i.e. >10 cm DHB; see Supporting Information—Fig. S1) demographic rates, we used two datasets belonging to the Guyafor network of forest permanent plots (https://paracou.cirad.fr/). The first dataset is based on eight permanent plots: one 25-ha and seven 6.25-ha plots (plots 9–15) set up between 1991 and 1992 to monitor the functioning and dynamics of both disturbed and undisturbed forests in Paracou (Gourlet-Fleury *et al.*, 2004). Between 1992 and 2015, censuses of all stems with a DBH > 10 cm were conducted every 5 years in the 25-ha plot, and every 2 years in the seven 6.25-ha permanent plots. The second dataset is from five 1-ha permanent plots established in undisturbed forests in Nouragues, a research station located *c*. 120 km from Paracou. In these plots, all stems with a DBH > 10 cm have been monitored at 2–4-year intervals since 1993.

To calculate sapling (1–5 cm DBH; see Supporting Information**—**Fig. S1) and juvenile (5–10 cm DBH; see Supporting Information—Fig. S1) demographic rates, we used 2 datasets: the first dataset belongs to the Guyafor network and is based on *c*. 750 8-m radii circular subplots established in 12 6.25-ha permanent plots to monitor forest regeneration dynamics in Paracou. Diameter increments and MR of saplings and juveniles have been monitored in these circular subplots at 3- or 5-year intervals between 1992 and 2016. The second dataset is from ten 20 × 250 m transects established in Paracou in 1994 by Molino and Sabatier (2001). In these transects, inventories of diameter growth and MR of saplings and juveniles were conducted for a 7-year period (1995–2002).

Diameter relative growth rates (RGR, mm·mm^−1^y^−1^) were calculated as ln(DBH_f_/DBH_i_)/Δ*t*, where DBH_f_ and DBH_i_ refer to final and initial diameters, respectively and Δ*t* is the time in years between the latest and earlier censuses. MR (% y^−1^) were calculated as 100 × (1−(*N*_f_/*N*_i_)) × 1/*t*, where *N*_i_ is the initial number of individuals, *N*_f_ is the number of survivors and *t* is the time in years between measurements (Sheil and May 1996). RGR and MR were calculated separately for sapling (1–5 cm DBH), juvenile (5–10 cm DBH) and adult wood (10–50 cm DBH). The number of individuals and census intervals used to calculate species demographic rates at each ontogenetic stage are shown in Supporting Information—Table S1.

### Statistical analyses

Trait–demography relationships, for each ontogenetic stage, were examined using pairwise Pearson’s correlations (*stast* library (version 4.4.0), *cor* function), with species as data points. We also used Pearson’s correlations to evaluate trait-trait associations, as well as the effects of WE on WSG-demography associations (see above). Growth-mortality, for each ontogenetic stage, was examined using linear regressions (*lme4* library (version 1.1-34), *lm* function). All statistical analyses were conducted in R version 4.3.0 (R Development Core Team, 2019).

## Results

Overall, there was substantial variation in mean trait values both among species and ontogenetic stages. Most species mean trait values were higher in adults than in juveniles or sapling wood, except for fibre fraction (*F*_F_) and vessel number (*V*_N_), which were higher in sapling wood. Overall, the ranges of variation in wood traits were higher for saplings than for adult or juvenile wood. Species mean RGR was higher for juveniles, while MR was higher for saplings (Table 2).

### Relationships between wood traits and MR

**Figure 2.**
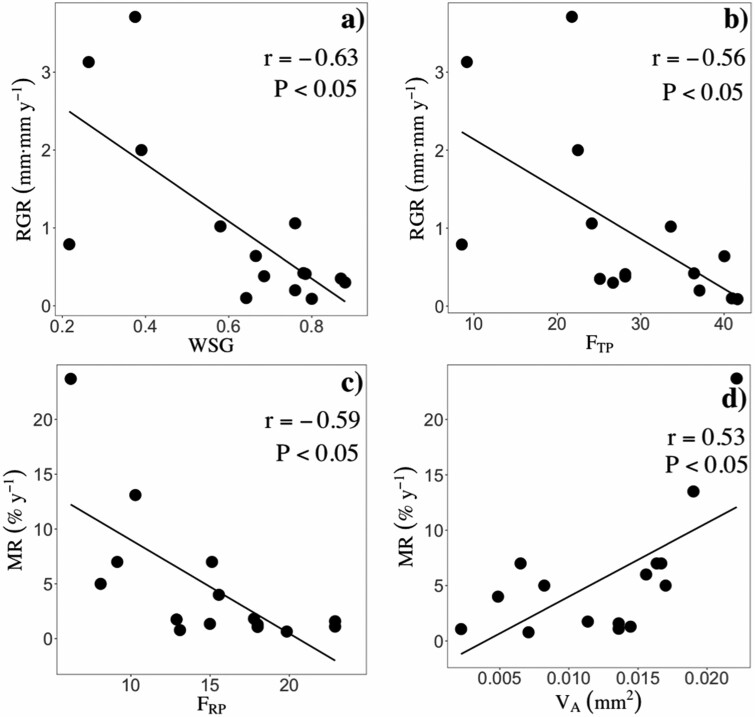
Significant pairwise relationships between demographic rates and wood traits at the sapling stage. A) WSG, B) total parenchyma fractions, C) radial parenchyma fractions and D) vessel lumen area. Points represent species means. Black and solid lines represent significant (*P* < 0.05) relationships. *P* values and Pearson correlation coefficients (*r*) are shown for each pairwise relationship.

**Figure 3.**
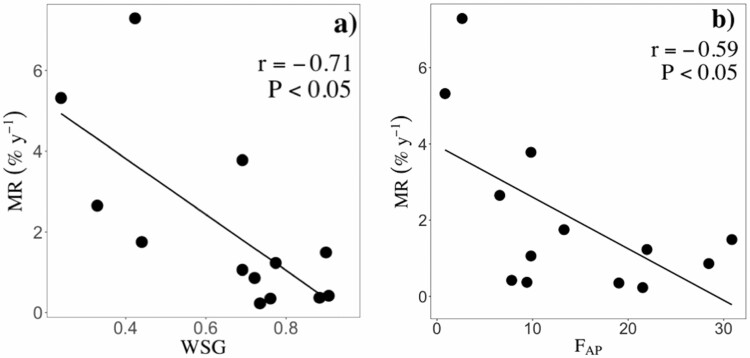
Significant pairwise relationships between MR and wood traits at the juvenile stage. A) WSG, and B) *A*_PF_. Black and solid lines represent significant (*P* < 0.05) relationships. Points represent species means. *P* values and Pearson correlation coefficients (*r*) are shown for each pairwise relationship.

Sapling and juvenile MR were significantly related to different wood traits (Table 3). In contrast, adult MR was unrelated to wood traits (Table 3). Sapling MR decreased with radial parenchyma fractions (*F*_RP_; Fig. 2c; *r* = −0.59, *P* < 0.05), but increased with vessel lumen area (*V*_A_; Fig. 2d; *r* = 0.53, *P* < 0.05); while juvenile MR decreased with both WSG (Fig. 3a; *r* = −0.71, *P* < 0.05) and axial parenchyma fractions (*A*_PF_: Fig. 3b; *r* = −0.59, *P* < 0.05).

**Table 3.** Pairwise Pearson correlations between wood traits and demographic rates for sapling, juvenile and adult wood. See Table 2 for trait abbreviations. Significant correlations (*P* < 0.05) are shown in bold.

Traits	Sapling wood		Juvenile wood		Adult wood	
	RGR	MR	RGR	MR	RGR	MR
**WSG**	**−0.63**	**−**0.41	**−**0.51	**−0.71**	**−**0.43	**−**0.15
***F***_**F**_	0.48	0.39	0.52	0.35	0.35	0.08
***F***_**TP**_	**−0.56**	**−**0.29	**−**0.48	**−**0.46	**−**0.28	**−**0.08
***F***_**AP**_	**−**0.39	**−**0.02	**−**0.25	**−0.59**	**−**0.07	**−**0.05
***F***_**RP**_	**−**0.32	**−0.59**	**−**0.43	0.19	**−**0.34	**−**0.06
***F***_**V**_	0.17	**−**0.31	0.29	0.4	**−**0.13	0.1
***V***_**A**_	0.49	**0.53**	0.38	**−**0.01	**0.46**	0.32
***F***_**WT**_	**−**0.26	**−**0.41	**−**0.12	**−**0.43	**−**0.27	0.17
**P**					**−**0.12	0.11
**Ca**					0.13	0.3
**K**					**0.47**	0.23
**Mg**					0.20	0.25

Note: Multiple comparisons corrections were not accounted for.

### Relationships between wood traits and diameter growth rates

**Figure 4.**
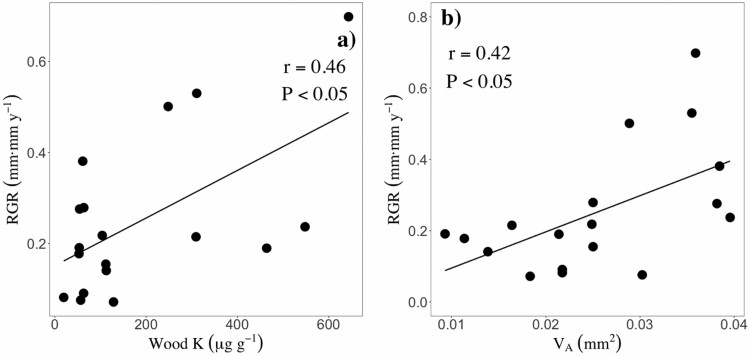
Significant pairwise relationships between growth rates and wood traits at the adult stage. A) wood potassium concentrations and B) vessel lumen area. Black and solid lines represent significant (*P* < 0.05) relationships. Points represent species means. *P* values and Pearson correlation coefficients (*r*) are shown for each pairwise relationship.

Sapling and adult relative growth rates (RGR) were significantly related to different wood traits, while juvenile RGR were unrelated to wood traits (Table 3). Sapling RGR were negatively related to both WSG (Fig. 2a; *r* = −0.63, *P* < 0.05) and total parenchyma fractions (*F*_TP_; Fig. 2b; *r* = −0.56, *P* < 0.05), while adult RGR scaled positively with *V*_A_ (Fig. 4a; *r* = 0.46, *P* < 0.05) and wood K concentrations (Fig. 4b; *r* = 0.47, *P* < 0.05).

### Effect of WE on WSG-demography associations

The concentration (i.e. % wood dry mass) of WE, in both heartwood and sapwood, is known for five of our study species (i.e. *Bocoa prouacensis*, *Virola michelii*, *Sextonia rubra*, *Bagassa guianensis* and *Dicorynia guianensis*). In these species, WE concentrations are low (<7%), and the differences between heartwood and sapwood WE concentrations are small (<3%, Rodrigues 2010; Amusant *et al.* 2014). The Pearson correlations that included WSG_REC_ (see ‘Materials and Methods’ section) did not differ, in terms of the correlation coefficients or level of significance, from the Pearson correlation that included WSG (data not shown).

## Discussion

### Relationships between wood traits and MR

We hypothesized that MR will decrease with stem traits related to wood strength (i.e. WSG, fibre fractions or FWT) and the fractions of either axial (AP) or radial (RP) parenchyma. In line with this expectation, we found that juvenile MR were negatively related to WSG (Fig. 3a; *r* = −0.71, *P* < 0.05). This agrees with a number of studies showing a negative relationship between WSG and juvenile MR in closed-canopy tropical forests (e.g. Wright *et al.* 2010; Phillipson *et al.* 2014). Different and non-exclusive reasons may explain this finding. First, in the shaded understory, where carbon gain is limited by low light availability, selection should favour denser tissues that increase survival due to reduced mechanical damages from falling debris or lower risk of pathogen attacks (Alvarez-Clare and Kitaima 2007; Kitajima *et al.* 2012; Reich 2014). For instance, stem tissue density was strongly correlated with the modulus of elasticity and toughness of stems, and was the best predictor of species MR in saplings of eight lowland tropical tree species (Alvarez-Clare and Kitajima 2007). Second, high-WSG species may have lower MR because they can be more tolerant to drought events. For example, at Paracou, high-WSG species tend to have lower xylem turgor loss points than low-WSG species, which may reduce the detrimental effects of water deficits on living cells (Santiago *et al.* 2018). Third, as both *F*_WT_ and *T*_PF_ scale with WSG at the juvenile stage [Supporting Information—Fig. S3b], it is possible that these traits might mediate the negative association between WSG and juvenile MR, as thicker fibres increase stem strength and living parenchyma cells can provide an active response against pathogens (Morris *et al.* 2016*b*). Further studies could examine the mediating effects of wood anatomical traits on the links between WSG and plant performance.

We also found a significant and positive relationship between vessel lumen area (*V*_A_) and sapling MR (Fig. 2d; *r* = 0.53, *P* < 0.05). This finding agrees with that of Osazuwa-Peters *et al.* (2017), who showed that sapling MR increased with vessel lumen size in a semi-deciduous forest in Panama. According to the ‘rare pith hypothesis’, wider conduits can have more interconduit pits and pit membranes and, therefore, should be potentially more vulnerable than narrow conduits to air-seeding through pits (Wheeler *et al.* 2005). Therefore, one possible explanation for this result should be that species with wider conduits have a higher risk of drought-induced xylem embolisms, which may eventually cause mortality. Yet, we ruled out this possibility, as at our study site vessel lumen size was found to be independent from pit traits, and is weakly associated with xylem cavitation (Levionnois *et al*., 2020). Instead, we suggest that the observed association between *V*_A_ and MR, at the sapling stage, can reflect allocation and demographic differences between species. Fast-growing species typically have wider conduits because they favour water transport and ultimately growth (e.g. Santiago *et al.* 2004; Russo *et al.* 2010; Hietz *et al.* 2016). Yet, at the same time, species that grow faster tend to have higher MR (e.g. Poorter *et al.* 2008; Russo *et al*., 2020). For instance, among our study species, growth rates were traded-off against MR at the sapling stage [Supporting Information—Fig. S2a]. Evidence suggests that saplings of fast-growing species have higher MR because they tend to have poorly defended tissues (Alvarez-Clare and Kitajima 2007; Kitajima *et al.* 2012). It is likely, then, that saplings with wider conduits (i.e. fast-growing saplings) die more often because they are more susceptible to pathogens or herbivores (e.g. Alvarez-Clare and Kitajima 2007; Zhu *et al.* 2018), rather than as a consequence of hydraulic failure.

In line with our expectation, sapling and juvenile MR were negatively related to radial (*F*_RP;_Fig. 2c; *r* = -0.59, *P* < 0.05) and axial (*F*_AP_; Fig. 3a; *r* = −0.59, *P* < 0.05) parenchyma fractions, respectively. Storage of nutrients and NSC (i.e. starch, soluble sugars and lipids) is thought to be one of the main functions of xylem parenchyma cells (e.g. Morris *et al.* 2016a). For example, both axial and radial parenchyma fractions have been positively related to the concentrations of NSC (e.g. Plavcová *et al.* 2015; Godfrey *et al.*, 2019). Therefore, a higher allocation of wood volume to *F*_RP_ or *F*_AP_ might enable higher storage of NSC, which, in turn, may increase survival because NSC favour a faster recovery from periods of drought, defoliation or shading (Poorter and Kitajima 2007; Herrera‐Ramírez *et al*., 2021). An alternative, but not mutually exclusive, explanation is that radial and axial parenchyma cells increase survival, as they play a critical role in plant defense by providing an active response against xylem infections and mechanical damages (Morris *et al.* 2016*b*). Although *F*_RP_ and *F*_AP_ were significantly related to sapling and juvenile MR, parenchyma fractions were unrelated to MR at the adult stage. This may be due to the possibility that other parenchyma traits, such as spatial arrangement and morphology, can have effects on tree functioning, in addition to the cross-sectional fractions measured here. For instance, axial parenchyma associated with vessels (i.e. paratracheal AP) is suggested to be of central importance in hydraulic balance (e.g. Morris *et al.* 2017; Aritsara *et al.*, 2020), while banded axial parenchyma (i.e. AP arranged in bands) may play a role in limiting the spreading of decay (Morris *et al.* 2016, 2019). Moreover, the morphology of radial parenchyma cells (i.e. uniseriate or multiseriate rays) may affect stem hydraulics and, to some extent, mechanics (e.g. Zheng and Martinez-Cabrera 2013). Thus, future studies should consider measuring the spatial arrangements and classify parenchyma cells into more specific categories.

### Relationships between wood traits and RGR

As expected, adult RGR scaled positively with wood K (Fig. 4a, *r* = 0.47, *P* < 0.05). This result is in line with fertilization experiments in lowland tropical forests showing that tree growth is strongly limited by soil K availability, although differed from those of Heineman *et al.* (2016), which reported no association between wood K and RGR. Potassium is suggested to play an important role in wood formation, mainly during cell expansion and osmoregulation (Ache *et al.*, 2009; Fromm 2010). Specifically, K is thought to be a driver of vessel formation (Fromm 2010), and studies using secondary ion mass spectrometry have reported higher concentrations of K in vessels than in other xylem cells (e.g. Langer *et al.* 2002). As fast-growing species need large vessels to increase xylem hydraulic conductivity and sustain high growth rates (e.g. Hietz *et al.* 2016), it is likely that they allocate proportionally more K to grow vessels than slow-growing species.

We found that *V*_A_ was positively associated with adult RGR (Fig. 4b; *r* = 0.46, *P* < 0.05). This result is in agreement with our hypothesis and with the results of several other studies (Russo *et al.* 2010; Fan *et al.* 2012; Hietz *et al.* 2016) which indicate that species with wider vessels grow faster. As xylem-specific hydraulic conductivity (Ks) increases exponentially with vessel lumen area, but only linearly with *V*_N_ (Tyree and Zimmermann 2002), Ks is expected to be significantly higher in species that build large conduits. In turn, Ks tends to be positively related to stomatal conductance and leaf maximum photosynthetic carbon gain (Santiago *et al.* 2004), which ultimately favours diameter growth rates (Poorter *et al.* 2010; Russo *et al.* 2010). We also predicted that RGR would decrease with traits related to stem structural strength (i.e. WSG, *F*_F_ or *F*_WT_). Consistent with this prediction, we found that sapling RGR was negatively related to WSG (Fig. 2a; *r* = −0.63, *P* < 0.05), which agrees with other studies in tropical forests (Poorter *et al.* 2008; Wright *et al.* 2010). One main reason to explain this result is the fact that, in sapling wood, species with high WSG have thicker fibre walls (Supporting Information**—**Fig. S3a), representing higher stem construction costs.

Total parenchyma fractions (F_TP_) were negatively related to sapling RGR (Fig. 2b; *r* = −0.56, *P* < 0.05). We are aware of only one study that has shown a significant, although positive, link between *F*_TP_ and RGR (Poorter *et al.* 2010). Parenchyma cells represent the majority of living cells in wood (Morris *et al.* 2016), besides living-fibres (Carlquist 2015). Living parenchyma cells, and, in particular, contact cells (i.e. cells having functional connections with vessels), are metabolically highly active (Spicer and Holbrook 2007). Therefore, although living parenchyma have been linked to several simultaneous functions such as storage, defense and transport (e.g. Pfautsch *et al.* 2015, Morris *et al.* 2016, 2019), they may also represent a significant maintenance cost for trees. Thus, stem maintenance costs could be mediating the negative relationship between *F*_TP_ with growth RGR in adult wood (Fig. 4, Table 3).

### Changes in trait-demography relationships during tree development

A number of studies have shown that both traits (Hietz *et al.* 2016; Rungwattana and Hietz 2017) and demographic rates (Wright *et al.* 2010; Hérault *et al.* 2011) can vary considerably during tree development. Thus, we expected that the trait–demography relationships may change accordingly. In line with this expectation, we found that WSG was negatively associated with MR only at the juvenile stage (Fig. 2d; *r* = −0.71, *P* < 0.05). High-WSG may represent a competitive advantage for individuals growing in the shaded understory (see discussion above), but not for adult trees that have reached the sunlit canopy. At Paracou, adult tree mortality tends to increase during the rainy season when storms are common (Aubry-Kientz *et al.* 2015; but see Pillet *et al.* 2018), and is also strongly associated with soil topography and drainage, with higher treefall rates in bottomlands with waterlogged soils where root anchorage is limited (Ferry *et al.* 2011). It is reasonable to expect, then, that traits determining tree biomechanical stability, such as rooting depth, crown architecture or the presence of buttressed roots may be better predictors of adult tree mortality in Paracou than WSG.

The Pearson correlation coefficients of the growth–trait relationships tended to be higher at the sapling compared to the adult stage (Table 3; Figs 2 and 3). This, together with the fact that adult MR were unrelated to wood traits, suggests that wood traits tended to be weakly related, or unrelated, to demographic rates at later ontogenetic stages. This agrees with previous findings at local (Visser *et al.* 2016; Osazuwa-Peters *et al.* 2017) and regional scales (Poorter *et al.* 2008; Wright *et al.* 2010). One possible factor explaining this result is the strong gradient in light availability that characterizes our study site (e.g. Baraloto *et al.* 2005; Laurans *et al.* 2012). In closed-canopy forests, light availability is an important factor mediating morphological and demographic variations across species, because it limits carbon gain (Wright *et al.* 2010). In general, saplings growing in the understory experience lower and more variable light availability than adult trees that grow in the canopy or subcanopy (Wright *et al.* 2010). As a consequence, interspecific differences in traits and demographic rates are expected to be higher in saplings than in adults (e.g. Poorter *et al.* 2010). Among our study species, the range of variation of most traits, as well as of MR, is higher for saplings than for juveniles or adults (Table 2). This may have favoured the detection of stronger relationships between traits and demographic rates at early ontogenetic stages. Another factor that may contribute to the observed changes in the strength of the trait–growth relationships during tree development may be related to size-related shifts in growth rates. For instance, for many tree species in Paracou, growth rates are lower at larger than at intermediate stem diameters (Hérault *et al.* 2011), possibly due to a shift in resource allocation from diameter growth to reproduction (Thomas 1996).

A large body of literature has shown that WSG is in general a good proxy of growth and MR (e.g. Poorter *et al.* 2008; Wright *et al.* 2010; Hérault *et al.* 2011). Yet, our results indicate that the associations between WSG and demographic rates are not always supported for different ontogenetic stages. For instance, at the sapling stage, WSG was negatively related to RGR, but unrelated to sapling MR; while the opposite trend was found at the juvenile stage. These findings add to recent studies showing that demographic rates may be better explained in some cases by wood anatomical (Poorter *et al.* 2010; Russo *et al.* 2010; Fan *et al.* 2012; Osazuwa-Peters *et al.* 2017) or chemical traits (Martin *et al.* 2014) than by WSG. This may be due to the fact that these traits are more closely related to performance than WSG. For example, vessel lumen area is more directly related to water transport capacity, and consequently to growth rates, than WSG (e.g. Fan *et al.* 2012). Furthermore, species with similar values of WSG may be functionally different, because similar values of WSG may be product of different wood anatomies (Zieminska *et al.* 2015).

Finally, it is important to note that while our study species reflect a wide spectrum of ecological strategies and wood structures, the number of species we sampled is relatively low, which might limit the generalizability of our findings. Thus, we suggest that further studies should examine whether the trait-demography associations reported in this study are consistent for larger sets of species and for different forest types as well.

## Conclusions

In this study, we examined trait-demography associations of sapling, juvenile and adult wood of 19 tree species from eastern Amazonia. We found that, in general, demographic rates of saplings, juveniles and adults were associated with different wood traits. We also showed that the strength of the trait-demography associations tended to decrease at later ontogenetic stages. Overall, our results support the growing evidence that the effects of traits on species demographic rates can change during tree development. Moreover, our findings indicate that WSG may not always be a good proxy of growth and mortality, particularly when traits such as wood chemical or anatomical traits are considered. These findings also support the general expectation that traits can be strongly related to species demographic rates and, hence, that traits are important to increase our understanding of life-history variations and community dynamics in lowland tropical forests.

## Supporting Information

The following additional information is available in the online version of this article –

**Table S1**. RGR and MR for saplings, juveniles and adults of 19 tree species from a lowland forest in eastern Amazonia.

**Figure S1.** Schematic representation of trait and demographic sampling.

**Figure S2**. Relationships between relative growth and MR for tree species in a lowland forest in eastern Amazonia.

**Figure S3**. Correlation matrices, using Pearson correlation coefficients, among wood traits.

plad090_suppl_Supplementary_Figures_S1-S3_Tables_S1

## Data Availability

The data that support the findings of this study are openly available in Figshare.com at: 10.6084/m9.figshare.22015805.
