# Supplementary material for: The links between wood traits and species demography change during tree development in a lowland tropical rainforest
Source: AoB Plants. 2023 Dec 27;16(1):plad090. doi: 10.1093/aobpla/plad090 (PMC10799319; doi:10.1093/aobpla/plad090)
Supplement: plad090_suppl_Supplementary_Figures_S1-S3_Tables_S1 [file plad090_suppl_supplementary_figures_s1-s3_tables_s1.pdf]

Supporting Information

Table S1. Relative growth rates (RGR, mm·mm^{-1} y^{-1}) and mortality rates (MR, % y^{-1}) for saplings, juveniles and adults of 19 tree species from a lowland forest in eastern Amazonia. Sample sizes (n), years of measurement (years), and datasets (data, see Material and methods) used to calculate demographic rates are shown for each species.

**Species**	**Saplings**				**Juveniles**				**Adults**			
	**Growth**		**Mortality**		**Growth**		**Mortality**		**Growth**		**Mortality**	
	RGR	n/years/data	MR	n/years/data	RGR	n/years/data	MR	n/years/data	RGR	n/years/data	MR	n/years/data
Bagassa guianensis									3.81	12/25/2nd	0.18	12/25/2nd
Cecropia obtusa	3.13	10/7/4th	23.7	40/7/4th	2.57	27/7/4th	2.65	35/7/4th	5.30	14/5/1st	7.27	19/23/1st
Dicorynia guianensis	0.10	20/24/3rd	13.1	307/7/3rd	0.83	30/5/3rd	0.86	17/6/3th	2.76	87/23/1st	0.66	147/23/1st
Eperua falcata	0.38	105/24/3rd	1.59	188/7/4th	0.93	26/5/3rd	1.06	69/7/4th	1.91	46/23/1st	0.46	105/23/1st
Eperua grandiflora	0.41	67/24/3rd	1.10	40/7/4th	0.45	12/24/3rd	3.78	57/5/3th	2.79	110/23/1st	0.02	147/23/1st
Eschweilera coriacea	1.02	43/6/4th	1.75	43/7/4th	0.95	12/6/4th	1.23	12/7/4th	1.55	146/23/1st	0.21	525/23/1st
Eschweilera sagotiana	0.64	136/25/4th	0.78	167/7/4th	0.93	50/24/4th	0.23	61/7/4th	1.41	245/23/1st	0.16	262/23/1st
Hirtella glandulosa	1.06	10/7/4th	1.35	11/7/4th					1.78	30/25/2nd	0.01	544/25/2nd
Lecythis persistens	0.20	74/5/3rd	0.75	271/7/4th	0.83	176/5/3rd	0.35	122/7/4th	0.91	251/23/1st	0.14	571/23/1st
Licania alba	0.35	19/5/3rd	1.29	172/23/4th	0.77	23/5/3rd	0.42	68/7/4th	0.82	109/23/1st	0.68	559/23/1st
Miconia tschudyoides	3.71	51/7/4th	1.32	503/7/4th	1.8	254/7/4th	1.75	86/7/4th	1.90	14/7/1st	0.21	40/5/1st
Oxandra asbeckii	0.30	99/5/3rd	1.07	328/5/3rd	1.13	41/5/3rd	0.37	154/5/3th	0.72	117/23/1st	0.78	733/23/1st
Parkia nitida			3.13	11/7/4th					6.98	10/10/1st	0.12	17/23/1st
Parkia velutina									5.01	11/23/1st	0.12	16/23/1st
Recordoxylon speciosum	0.09	12/24/3rd	2.71	40/7/4th	0.39	11/7/4th	1.49	11/5/3th	2.18	28/23/1st	0.44	32/23/1st
Schefflera morototoni	0.79	14/24/3rd	1.66	54/7/4th	0.98	16/7/4th	5.32	22/7/4th	4.3	12/23/1st		
Sextonia rubra	0.42	19/5/3rd	1.82	33/7/3rd			2.57	18/7/3rd	2.15	101/23/1st	0.99	108/23/1st
Swartzia panacoco	0.83	11/7/4th							0.76	14/23/1st	0.58	24/23/1st
Virola michelii	0.48	138/24/3rd	0.65	111/7/4th	0.48	19/24/3rd	7.29	22/5/3rd	2.37	61/23/1st	1.76	79/23/1st

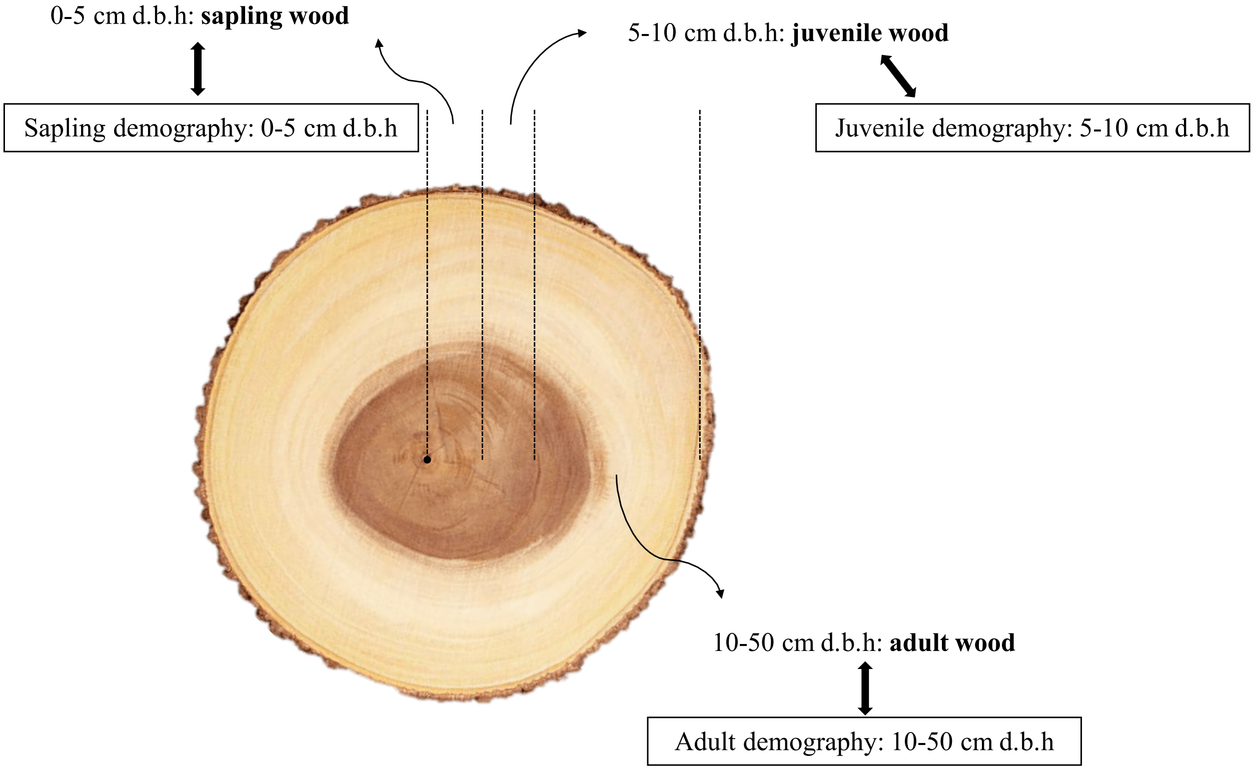

Fig. S1. Schematic representation of trait and demographic sampling. First, each stem disc was divided in three radial sections: sapling (0.5 cm d.b.h), juvenile (5-10 cm d.b.h) and adult wood (10-50 cm d.b.h). Then, traits were measured for sapling, juvenile and adult wood (see Methods for details), and species mean traits were related to corresponding species mean growth and mortality rates.

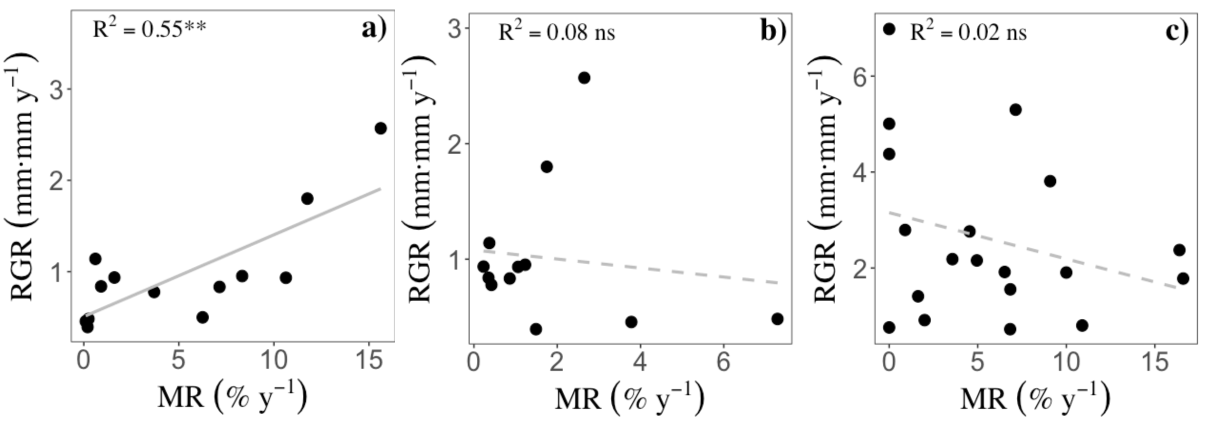

Fig. S2. Relationships between relative growth (RGR) and mortality rates (MR) for tree species in a lowland forest in eastern Amazonia. a) saplings, b) juveniles and c) adults. Coefficients of determination (R^2) and P values are given. Solid and dashed lines represent significant ($P<0.05$) and non-significant regression fits, respectively. Significant levels are: **: $P<0.01$, and ns: $P>0.05$.

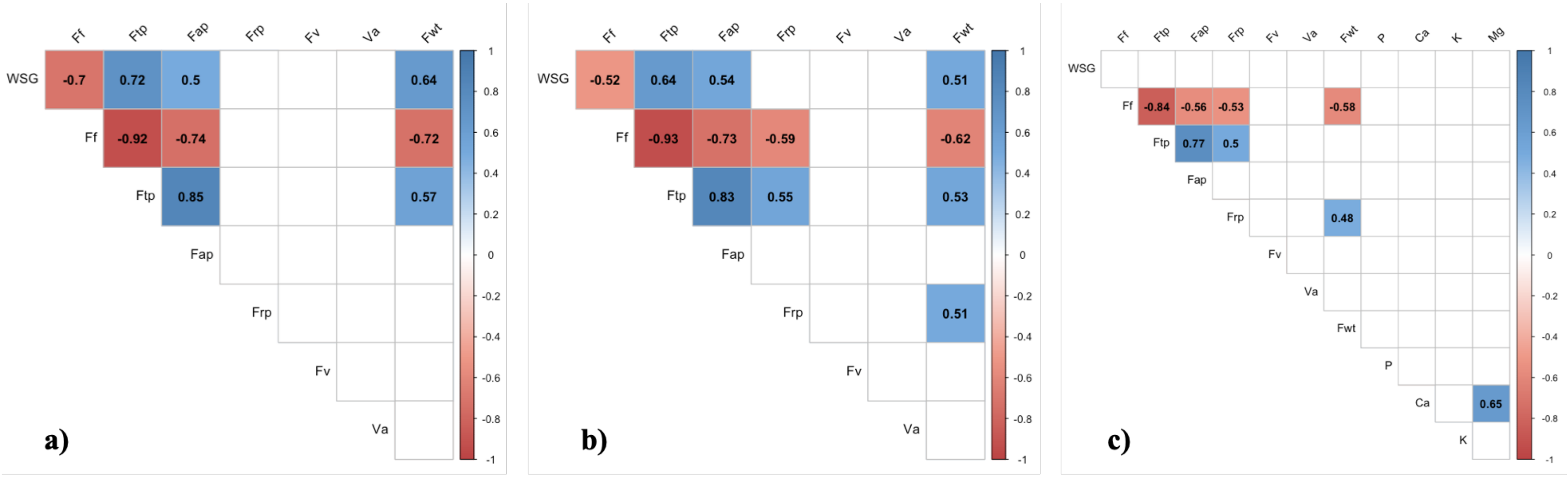

Fig. S3. Correlation matrices, using Pearson correlation coefficients, among wood traits measured on sapling (a), juvenile (b) and adult wood (c) of 19 tree species from a lowland forest in French Guiana. Significant correlations (P<0.05) are colored, where blue shades represent positive correlations and red shadows negative ones. See Table 2 for trait abbreviations.
